# Update on the Biological and Clinical Relevance of Mast Cells in Chronic Rhinosinusitis with Nasal Polyps

**DOI:** 10.3390/biomedicines12112647

**Published:** 2024-11-20

**Authors:** Luca Giovanni Locatello, Silvia Tonon, Vincenzo Mele, Simone Santini, Cesare Miani, Carlo Ennio Michele Pucillo

**Affiliations:** 1 Department of Otorhinolaryngology, Academic Hospital “Santa Maria della Misericordia”, Azienda Sanitaria Universitaria Friuli Centrale, Piazzale Santa Maria della Misericordia 15, 33100 Udine, Italy; 2 Department of Medicine (DMED), Immunology Section, University of Udine, Piazzale Kolbe 4, 33100 Udine, Italy; 3 Department of Medicine (DMED), University of Udine, Via Colugna 50, 33100 Udine, Italy

**Keywords:** nasal polyps, mast cells, biologics, rhinosinusitis, eosinophils

## Abstract

Chronic rhinosinusitis with nasal polyps (CRSwNP) is a common inflammatory disorder whose complex immunopathogenesis has yet to be fully elucidated. Endotype-2 CRSwNP is the most common form of disease where eosinophils are the main drivers of inflammation. Traditional treatments for CRSwNP have centered around intranasal or systemic corticosteroids and endoscopic sinus surgery (ESS). However, recent advancements in targeted therapies have introduced novel biological agents that specifically target key inflammatory mediators such as IL-4, IL-5, and IL-13. These biologics offer promising options for patients with CRSwNP, particularly those who do not respond adequately to conventional treatments. Nonetheless, some patients do not satisfactorily respond to these drugs because of an insufficient blockade of the inflammatory process. The mast cell (MC) is another important (and somehow neglected) actor in the pathogenesis of CRSwNP, and the latest clinical and translational evidence in this field has been reviewed in the present paper.

## 1. Introduction

CRSwNP is a chronic inflammatory condition affecting the nasal and sinus cavities that, by definition, is accompanied by the growth of nasal polyps. Despite extensive research, the underlying mechanisms driving this disorder remain a complex puzzle, highlighting the urgent need for a more comprehensive understanding [1]. In the effort to unravel the multifaceted nature of CRSwNP, researchers have identified three distinct endotypes based on the predominant inflammatory cells and mediators involved: Type 1 (TH1-driven), Type 2 (TH2-driven), and Type 3 (TH17-driven) [2,3].

Type 2 CRSwNP, mainly characterized by the infiltration of eosinophils, is the most prevalent form of the disease, and this holds particularly true in Western populations [4]. While traditional treatments such as intranasal corticosteroids and endoscopic sinus surgery have been effective for many patients, the search for more targeted and effective therapies has led to the development of novel biological agents that specifically target T2 inflammation [5]. However, the optimal treatment approach for CRSwNP remains a clinical challenge, as many patients exhibit mixed inflammatory phenotypes (e.g., type 2/1; type 2/3) and they may not respond adequately to currently available therapies [6,7,8].

Mast cells (MCs), key immune cells involved in tissue inflammation and remodeling, have emerged as significant players in the pathogenesis of CRSwNP. MCs are classically divided into two major phenotypes: “mucosal” MCs, which mostly produce tryptase (MCT), and “connective” MCs that synthesize tryptase/chymase (MCTC) [9]. Recent studies have also identified distinct MC phenotypes in nasal polyps, including a unique subepithelial population that is enriched in T2 microenvironments [9]. These findings suggest a crucial role for MCs in the immunopathogenesis of CRSwNP and highlight their potential as promising therapeutic targets.

Given the persistent nature of CRSwNP and the limitations of current treatment options, there is a pressing need for innovative approaches that address the underlying mechanisms of the disease. By elucidating the role of MCs and other key immune cells, researchers may be able to develop more targeted and effective therapies for patients with CRSwNP, improving their quality of life and reducing the burden of this chronic condition.

## 2. Materials and Methods

**Figure 1:**
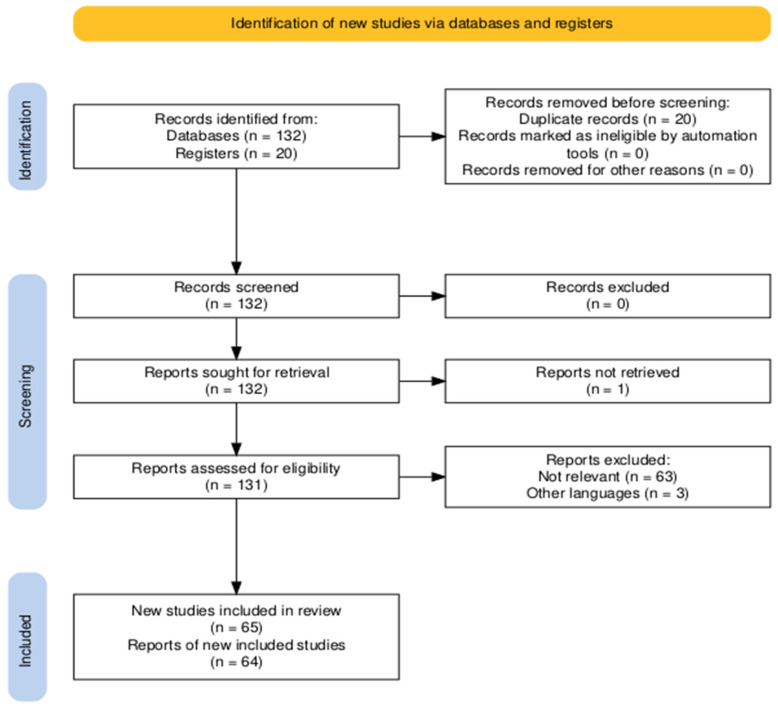
PRISMA flowchart for the selection of the articles discussed in the present review.

The PRISMA statement was followed in the preparation of the present paper, and a modified PRISMA flowchart is shown in Figure 1 [10]. The figure was generated using the freeware and web-based Shiny application provided by the Haddaway group [11]. No institutional review board approval was required for the present work. The present review has been registered on the Open Science Framework—OSF Public Registry with the registration DOI 10.17605/OSF.IO/MB2Z3 (Center for Open Science, Charlottesville, VA, USA; the whole protocol can be found at https://osf.io/mb2z3, accessed on 16 November 2024).

The MEDLINE PubMed and Cochrane Library databases were used to perform the literature search, from 1 January 2014 to 1 November 2024. The following search strings were used: “(CRSwNP OR nasal polyps OR nasal polyposis) AND (mast cells OR mastocyte)”. All the relevant articles, that is, those presenting new original clinical, basic, and translational data about the role of MCs in CRSwNP, were included after careful reading of the titles and abstracts. The full texts of the included articles were then retrieved by the first author (LGL), and quantitative and qualitative data were synthesized accordingly. Articles were excluded if they were non-relevant (e.g., other systematic, scoping, narrative reviews in order to avoid repetitions and overlaps) or off-topic papers (Reason 1); or if they were written in languages other than English (Reason 2).

The search strategy retrieved a total of 152 articles, and, after applying the selection criteria, a total of 62 full texts were finally analyzed. Duplicates were removed using Mendeley Reference Manager (version 2.97.0, © 2023 Mendeley Ltd., © 2024 Elsevier Ltd., 1043 NX Amsterdam, The Netherlands), and additional 3 articles (for a total of 65) were retrieved and included in the discussion after checking through the reference lists of the relevant studies. Quantitative and qualitative data on surgical outcomes were summarized and systematically presented in tables.

## 3. Results

### 3.1. Evidence of Mast Cell Involvement in the Pathogenesis of CRS in Non-Human Models

The exact cause of CRSwNP remains elusive, and non-human models remain important to obtain robust experimental data. Regarding MCs, Hua et al. compared *C57BL/6 wt* and *C57BL/6-Kit* (W-sh/W-sh) MC-deficient mice after intraperitoneal and allergen exposure; tissue eosinophilia and mucosal goblet cell hyperplasia were significantly reduced in the latter population compared to the *wt*, while none of the MC-deficient mice developed polypoid degeneration of the sinonasal tract [12]. In a mouse model of eosinophilic (herein considered a surrogate for endotype 2) CRSwNP, exposure to cigarette smoke has been demonstrated to significantly augment the number of polyp-like lesions. Of note, this increase is accompanied by an increase in MC numbers [13].

Recently, a role for some bacteria in the development of CRSwNP, and in particular *Staphylococcus aureus*, has been hypothesized. It has been demonstrated that the intranasal challenge with *S. aureus* biofilm-secreted factors isolated from patients with CRSwNP induces an increase of MCs in the nasal mucosa of rats [14]. In a laboratory mouse strain, constant exposure to house mite was associated with abundant MCs in nasal mucosa, while exposure to the combination of house mite plus *S. aureus* enterotoxin B (SEB) increased eosinophilic infiltration in polypoid lesions [15]. Another research paper has instead investigated if there are some strain-specific factors that may explain this relationship; in a cohort of 72 CRS (with and without NP) patients, *S. aureus* harboring the virulence genes *lukF.PV* (Panton–Valentine leukocidin), *sea* (staphylococcal enterotoxin type A), and *fnbB* (fibronectin-binding protein B) were isolated in excised tissue samples that showed higher MC frequencies [16]. Finally, in other papers, the functional loss of periostin (a clinical biomarker of injury or inflammation that can be measured also in blood) seemed to enhance the formation of polyp-like lesions as well as to favor MC recruitment in a mouse model of eosinophilic CRSwNP [17,18]. Therefore, non-human models are still useful in finding potential new molecular targets for this inflammatory condition.

### 3.2. Laboratory Methods to Study Mast Cells in Nasal Polyps

Human nasal polyps can be easily obtained by surgical procedures or in-office endoscopic-assisted biopsy; these procedures also permit to obtain “healthy” control tissue from the same patients, usually from the uncinate process or the inferior turbinate. In addition to histochemical methods, single-cell RNA sequencing (scRNA-Seq), flow cytometry, or in vitro culture can be used to study the immunological basis of CRSwNP. A recent publication has compared different methods of handling polyp samples to minimize batch effects. The authors conclude that for studies investigating sinonasal epithelial cells, the cryopreservation of tissue (of approximately 3 mm^3^) in CS10 medium may be preferred, whereas for studies focusing on MCs, cryopreservation of cell suspensions may be viewed as the best method available at present [19]. Finally, a comprehensive and detailed protocol for MC isolation, digestion of nasal polyps, and characterization by intracellular protease immunostaining has been recently published by Derakshan and Dwyer (which the interested readers are referred to) [20].

### 3.3. MCs as Drivers of Mucosal T2 Inflammation in CRSwNP

Eosinophils are thought to be the key cells underlying type 2 inflammation in CRSwNP, but the underlying pathophysiology remains incompletely understood [1,5]. Type 2 cytokines such as IL-4, IL-5, and IL-13 (i.e., the targets of the newly approved monoclonal antibodies for uncontrolled severe and refractory CRSwNP) are derived not only from eosinophils but also from other cellular subsets, including innate lymphoid cells 2 (ILC2), basophils, and MCs [5]. Historically, the presence of MCs in nasal polyps was noted many decades ago [21], but the research interest in this area stemmed from the seminal paper of Takabayashi et al. [22]. The group collected nasal tissues and lavage fluids from patients with CRS and controls (non-CRS patients and tissue from the uncinate process of the same patient). They showed a significant abundance of MC_T_s in the epithelium and of MC_TC_s in the glands of nasal polyps. Such MC populations were functionally active, with elevated tryptase levels in nasal lavage fluids. In addition, a strong correlation between the levels of tryptase and eosinophil cationic protein (ECP) in nasal tissue extracts was shown, suggesting that MCs and eosinophils may use the same molecular pathways to recruit and activate each other [22]. The reciprocal interactions between MCs and eosinophils have been well characterized, and their common activating factor, interleukin-5 (IL5), is released by both cell types [23]. Over the last decade, many studies have identified a list of other activating and inhibitory molecules and pathways for MCs in CRSwNP, and a summary of these factors is given in Table 1 [24,25,26,27,28,29,30,31,32,33,34,35,36,37,38,39,40].

**Figure 2:**
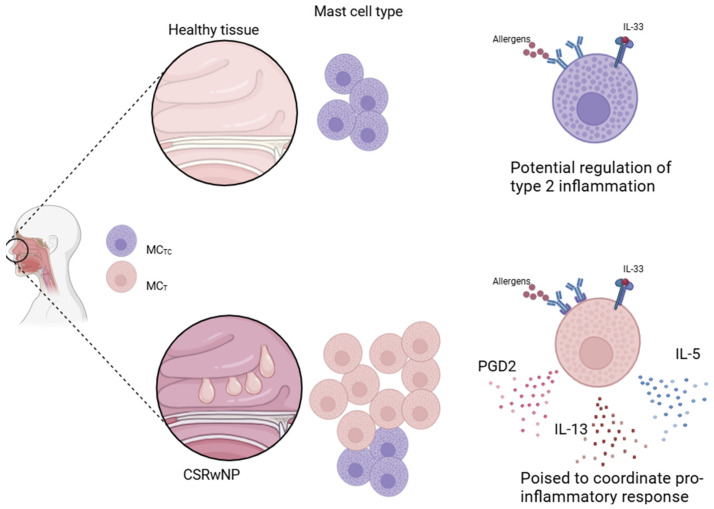
Based on single-cell data, it has been shown that healthy nasal tissue is characterized by the presence of MC_TCs_ (MC tryptase and chymase positive) with the potential to regulate type 2 inflammation, whereas in polyp tissue, MC_Ts_ (tryptase only) are also found. In particular, in this pathological setting, MCs are capable of producing pro-inflammatory cytokines such as IL-5 and IL-13, and also PGD2. In both contexts, MCs can be activated by the alarmin IL-33 or by IgE-mediated antigen stimulation.

Indeed, the use of single-cell technologies (that is, the multi-omics analysis of the transcriptome, metabolome, proteome, …) allowed great advances in the field of immunobiology, enabling the characterization of rare cell populations that were otherwise underestimated because of technical reasons. As for the role of MCs in CRSwNP, we found only four works based on these technologies (Table 2): two based on Seq-well [9,41] and two based on 10x Genomics technology [42,43]. In general, scRNA-seq analysis confirmed the presence of MCs in the nasal tissues with polyps and that their expansion is proportional to disease severity. Ordovas-Montanes et al. compared the ethmoid sinus with and without polyps and found out that MCs are specifically enriched in genes involved in prostaglandin D2 (PGD2) synthesis. Moreover, a great number of MCs did express IL-5 and IL-13 [41]. Three years later, the same group of authors identified four subclusters of MCs, all expressing tryptase and chymase, as well as enzymes involved in PGD2, histamine, and lipid mediators’ synthesis [9]. They focused their attention on the two more polarized subsets: MC1 and MC3. MC1 belongs to the MC_TC_ population, while MC3 belongs to the MC_T_ cluster. MC1 expresses a gene signature specific for IgE- and IL-33 activation, suggesting that these cells can respond to both innate and adaptive stimuli. On the other hand, MC3 expresses IL17RB, which is the receptor of IL-25, a cytokine involved in T2 inflammation. By also combining a CITE-seq analysis, they identified CD38 as a novel marker to distinguish MC3 from MC1; CD38 expression is reduced in MC3. Interestingly, all these MC subsets are positive for Ki67, with a major expression in the CD38^high^ population, indicating a proliferation capacity in situ, which can explain MC hyperplasia in nasal polyps [9]. One year later, Wang and coauthors identified two different subsets of MCs: one MC_T_ APOE^+^ and one MC_TC_ [43]. According to their data, MC_Ts_ are expanded in eosinophilic CRSwNP patients, while MC_TCs_ are more abundant in healthy controls (Figure 2).

Interestingly, the MC_T_ APOE^+^ subpopulation was poised to produce prostaglandin D2, leukotrienes, histamine, and nitric oxide [43]. The authors did not discuss this discordance with previous findings, but we need to consider that Dwyer et al. studied the intra-polyp variation of MCs while Wang et al. compared MCs among healthy controls, ethmoid sinus tissue with and without polyps [9,43].

Apart from eosinophils, the interactions between MCs and other cellular populations are beginning to be elucidated. For example, tuft cells (or solitary chemosensory cells) are abundant in NPs, and they are known to release IL-25 and Cys leukotrienes that can stimulate ILC2, MCs, and T2-skewed inflammation [44,45]. In another preliminary study, a group from the UK specifically investigated the expression of PGD2 and its receptor PTGDR2 in several samples from nasal polyps and inferior turbinates [46]. Somehow, in contrast to what other groups had found, [33] Xia et al. showed that only around 8% of MCs compared to 80% of circulating basophils expressed PGD2, and high levels of PTGDR2 mRNA were found only in basophils. Since basophils are rarely described in nasal polyps, these results should be considered with caution until new evidence is available [46]. MCs also interact with microbial cells, and, in 2015, a group of UK researchers first documented the residency of biofilm-forming *S. aureus* inside MCs [47]. In 2020, a subsequent study revealed how living bacteria entered the cytoplasm of MCs through phagocytosis, and this is made possible by exploiting the aforementioned SEB: endotoxin release by bacteria can recruit MCs, which in turn internalize and transport them to the subepithelial layer. Some of the intracellular bacteria are still able to proliferate until MC rupture; subsequent cellular lysis releases proinflammatory cytokines, and viable *S. aureus* causes epithelial damage, thus promoting an inflammatory environment that facilitates the growth of polyps [48]. These studies highlight the need for managing a potentially treatable trigger by disrupting or reducing the bacterial biofilm (local antibiotics, etc.).

### 3.4. MCs in the Pathophysiology of AERD

AERD, i.e., the association of CRSwNP, asthma, and acetylsalicylic acid (ASA) hypersensitivity, is a T2-skewed inflammatory syndrome, independently described by Fernand Widal and Max Samter in the last century. The pathophysiology of this condition is based on the disruption of the arachidonic acid metabolic pathway, but the exact cause remains elusive.

Several peculiar molecular changes in the sinonasal microenvironment have been identified in AERD. These inflammatory signatures include, for example, that NPs from AERD subjects have markedly increased expression of the alarmin-like cytokine IL-33 and that IL-33 is required for MC activation and bronchoconstriction [49]. Prostaglandin PGD2 is the major COX product of MCs in AERD and its associated T2 immune responses; the Buchheit group has demonstrated that its production is driven by the innate cytokine thymic stromal lymphopoietin (TSLP) [50]. However, eosinophils have also been shown to be an important source of PGD2 in AERD subjects [51]. Another research group has found specific transcriptomic differences between AERD and ASA-tolerant patients with the former group that showed significantly higher levels of IL-5 and CCL17 in nasal secretions. In the same study, MCs in the setting of AERD were shown to differentially upregulate some genes such as *IL17RB*, *VEGFA*, colony-stimulating factor 1 (*CSF1*), the nuclear enriched abundant transcript (*NEAT1*), and the 15-hydroxyprostaglandin dehydrogenase (*HPGD*) [52]. Beyond all the cytokines, Takahashi et al. showed that MCs are the preeminent cells in orchestrating the AERD inflammatory response. In their experiments, the authors used microparticles as a marker of cell activation, and it was demonstrated that epithelial barrier damage was worse and MCs were more activated in these patients compared to ASA-tolerant CRSwNP cases [53].

The interaction between nasal epithelial cells and MCs offers some clues to understanding this dysregulation: Stevens et al. showed that the epithelium of AERD cases overexpressed ALOX15 (15-lipooxygenase) compared to controls or non-AERD NPs. A mediator of ALOX15, 15-Oxo-ETE (15-oxo-eicosatetraenoic acid), was then shown to have the highest concentrations in AERD NPs. MCs expressing the aforementioned HPGD were localized near ALOX^+^ epithelium, and HPGD is a necessary enzyme for 15-Oxo-ETE synthesis [54]. In the end, the mechanistic dysregulation of the eicosanoids system remains puzzling, and much work is still to be performed; ifetroban, a thromboxane-prostanoid receptor antagonist, was used in a small trial on 35 patients with AERD [55]. Contrary to the expectations, the drug did worsen the sinonasal symptoms, and thus the authors hypothesized that TP signaling may maintain PGE2 production when COX-2 function is low [55].

### 3.5. Diagnostic and Prognostic Utility of MCs in CRSwNP

MCs cannot usually be found in peripheral blood while dosing plasmatic tryptase is performed only in systemic MC disorders. In the last decade, the interest in measuring tryptase in nasal lavage has not gained much interest apart from a few studies to be discussed. Groger et al. have found that levels of tryptase were significantly elevated in nasal discharges from allergic rhinitis patients, compared to CRS and controls; instead, eosinophilic cationic protein levels were significantly higher in NP compared to all other groups [56]. The same group has published another subsequent comparison study where higher levels of IL-5, IL-17, ECP, and tryptase, among others, were measured in NP compared to CRSsNP and healthy controls [57]. These authors recommended molecular tests as the first-line method to differentiate these conditions because they are “more comfortable”, but we strongly disagree with them given the sound role of endoscopic examination in every sinonasal pathology. More interestingly, Perić and coworkers have shown higher levels of nasal tryptase in AERD patients compared to simple CRSwNP, thus reaffirming the pivotal role of MCs in ASA intolerance [58].

MCs may also serve as prognostic and predictive factors in the treatment of NPs. For instance, a Japanese study has found a positive correlation between the membrane IgE-positive cells (counted on high-powered field histological preparations) and the radiological severity score of patients with CRSwNP [59]. In another small study, the number of MCs in resected polyps was negatively correlated with the risk of recurrence, estimated through the Japanese Epidemiological Survey of Refractory Eosinophilic Chronic Rhinosinusitis (JESREC) scoring system [60]. Again, after excluding AERD, MCs were more abundant in plasma cell-dominant CRSwNP patients (compared to eosinophil-dominant cases) in a paper by Lin et al., and subjective symptoms measured by the sinonasal outcome test 22 (SNOT-22) were worse in the former group [61]. T-cell/transmembrane immunoglobulin and mucin domain protein 3 (TIM-3) is a receptor that promotes MC activation (see Table 1). A 2021 study has found that higher MC levels were linked to earlier recurrence of sinonasal edema, but not with the need for future treatment with steroids post-operatively [62]. In another recent study, endotyping the immunoprofile in excised polyps identified the cluster of LTE4, PGD2, 15(S)-HETE, and IL-13 expression as the most likely to recur [63]. Interestingly, this cluster remains significant independently of aspirin tolerance, but a limit of this study is the lack of single-cell RNA sequencing; differentiating the role of MCs and eosinophils in these profiles remains at present impossible [63].

At present, we can conclude that the clinical and prognostic role of MCs in CRSwNP remains supported by weak and conflicting evidence. Another method to translate basic research into clinical practice might be offered by nasal cytology. For example, eosinophils and MCs are the major producers of the crystallized form of galectin-10, that is, Charcot–Leyden crystals. Recent work has correlated the presence of such crystals, which can be easily appreciated on cytological samples, with the severity of CRSwNP [64]. In another recent study involving 39 participants, cytology and immunohistochemistry of NPs were comparable in the measure of intraepithelial MCs (*p* = 0.002), and a histological cut-off of 6 intraepithelial MCs was identified to possibly detect severe CRSwNP (*p* < 0.001) [65]. In the end, a clinically relevant role of cytology in rhinology remains to be found, outside of experimental investigations [66].

### 3.6. Importance of MCs in the Era of Biologics for CRSwNP

**Figure 3:**
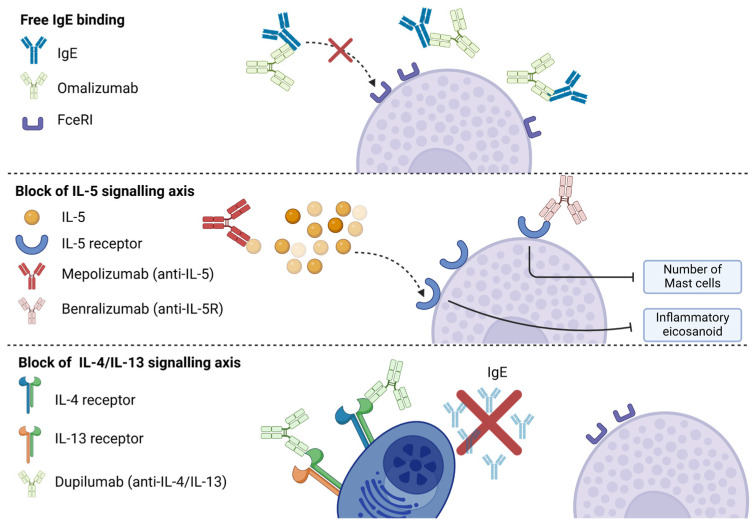
mAb for the direct or indirect interference of MC function in the context of CRSwNP. The activity of mast cells can be inhibited by preventing the binding of immunoglobulin E (IgE) to the Fc epsilon receptor 1 (FceRI). This can be achieved through the use of omalizumab, which binds directly to free IgE, or by preventing the production of IgE by plasma cells through the inhibition of interleukin 4 (IL-4) and IL-13 signaling. Additionally, the function of mast cells can be modulated by preventing the binding of IL-5 with mepolizumab or by directly blocking IL5R with benralizumab.

In the last decade, new drugs targeting T2 inflammation have entered clinical practice, as shown in Figure 3. These molecules have demonstrated significant results in controlling T2-skewed inflammatory disorders such as atopic dermatitis, eosinophilic esophagitis, eosinophilic granulomatosis with polyangiitis, and some types of CRSwNP and asthma. The efficacy of omalizumab (blocking the IgE-mediated activation of MC), mepolizumab (anti-IL-5), and dupilumab (anti-IL-4/IL-13) in improving CRSwNP endpoints is an indirect demonstration of the pathogenetic role of these cell subsets [67].

In more detail, mepolizumab has been shown to decrease the production of several eicosanoids (PGD2, PGF2α, and cysteinyl leukotrienes). In a study conducted on a small cohort of AERD patients, according to its authors, this would reflect the combined IL-5 signaling on local eosinophils, basophils, epithelial cells, and MCs [68]. The evidence remains only speculative, though, since serum tryptase levels were similar between patients on and off mepolizumab (*p* = 0.26), while nasal tryptase levels were not assessed [68]. Instead, a report on the use of reslizumab (another anti-IL-5) in an AERD patient showed that this drug depleted peripheral and NP eosinophils, but local MCs actually *increased* after starting the biologic drug. This might explain why the sinonasal symptoms of the patient worsened while the asthma control improved [69].

In a subanalysis of the OSTRO trial, benralizumab (directed against the IL-5R alpha subunit) instead reduced MC_Ts_ in nasal polyps but not in a statistically significant manner (from 39.8 to 9.6 cells/mm^2^ after 56 weeks of treatment versus a change from 33.2 to 29.1 cells/mm^2^ in the placebo group; *p* = 0.161) [70].

Regarding dupilumab, using the data from the SINUS-52 trial, Bachert et al. showed for the first time that the antibody decreased the density of MCs in nasal brushings, and patients under treatment showed reduced urinary LTE4 levels compared to placebo [71]. In another study involving 22 patients with AERD treated with dupilumab, many nasal mediators, including the pleiotropic cytokine oncostatin M produced by MCs, were reduced after three months of treatment. The authors concluded that IL-4Rα blockade, although primarily directed against eosinophils, may indirectly decrease mediators of innate inflammation and epithelial dysregulation, thus explaining the clinical efficacy of dupilumab in AERD [72]. In a recent series of fifty-two patients receiving dupilumab, a reduction of eosinophils in the nasal cytological infiltrate was observed after 6, 12, and 24 months, and a similar trend was demonstrated for MCs, although no formal statistics was used in these analyses [73].

Sialic acid-binding immunoglobulin-like lectin 8 (Siglec-8) is an important trans-membrane receptor that activates MCs and inhibits apoptosis in granulocytes. A few years ago, an intravenous infusion of anti-Siglec-8 antibody was used in a small clinical trial (ClinicalTrials.gov ID: NCT02734849). Unfortunately, the research was rapidly abandoned because its initial results were non-superior to placebo, despite the absence of any significant adverse reactions [74].

Finally, we must recall here that, among the previously cited molecules, the results of the NAVIGATOR trial on the use of tezepelumab (an antibody against TSLP) are promising for both asthma and CRSwNP, even though this drug is not at present approved for treating the latter condition [75].

## 4. Conclusions

Over the past decade, significant advancements have been made in our understanding of the role of MCs in the pathophysiology of CRSwNP. MCs, key immune cells involved in tissue inflammation and remodeling, have been implicated in various allergic and inflammatory diseases, including CRSwNP. Recent studies have identified distinct MC phenotypes in nasal polyps, suggesting their crucial role in the development and progression of this disorder.

While there are currently no approved drugs that directly target MCs, several of the existing biologics for nasal polyposis may indirectly reduce their proinflammatory activity in the nasal mucosa. These agents, primarily targeting T2 inflammation, may have beneficial effects on MC function by modulating the microenvironment and reducing the release of inflammatory mediators.

As our understanding of the molecular mechanisms underlying CRSwNP continues to evolve, future research may uncover novel therapeutic strategies that directly target MCs or their downstream signaling pathways. By targeting MCs, it may be possible to develop more effective and personalized treatments for CRSwNP, improving the quality of life for patients with this debilitating condition.

## Figures and Tables

**Table 1:** A synthesis of the latest findings on the activating and inhibiting pathways of MCs in CRSwNP in the last ten years. NPs, nasal polyps; FESS, functional endoscopic sinus surgery; mLKS, modified Lund Kennedy Score; HMC-1, human mast cell line.

Reference	Molecular Receptor(s) or Stimulus	Material/Sample	Technique Used in the Analysis	Main Findings
Bayar Muluk et al., 2021 [24]	Matrix metalloproteinase-2 (MMP-2) and MMP-9	Surgical samples of NPs	Immunohistochemical methods	Granulocyte and mast cell MMP2 and MMP9-PI were higher than the rate of monocyte MMP2-PI and monocyte MMP9-PI, respectively. Expression of MMP-2 and -9 may favor polypoid degeneration.
Compton et al., 2022 [25]	Substance P (SP), Interleukin 33 and 37 (IL33 and IL37)	Surgical samples of NPs versus controls during turbinoplasty and nasal lavages	ELISA, qPCR	Concentration of IL-33 was reduced in patients with NPs compared to controls. SP and IL-37 were higher in the lavage fluid of NP patients compared to controls.
Corredera et al., 2018 [26]	T cell immunoglobulin domain and mucin domain 3 (TIM-3)	Surgical samples of NPs	Flow cytometry for CD45, Live/Dead, c-kit, FcεR1, TIM-3	TIM-3+ MCs were present in the epithelium of NPs. In a subgroup of patients with concomitant asthma, increased epithelial TIM-3+ MCs were related to worse Lund Kennedy endoscopic scores. Oral corticosteroids did not affect the viability of mast cells nor their influence on the worse postoperative disease severity.
Derakshan et al., 2021 [27]	Subepithelial fibroblasts	Human umbilical cord blood-derived MCs (CBMCs) co-cultured with fibroblasts from CRSwNP and CRSsNP patients	Quantitative PCR, flow cytometric evaluation, and RNA sequencing	Subepithelial fibroblasts elicit CD69 upregulation in MCs, and this leads to overexpression of several molecules, including IL6, LIF, OSM, IL4R, etc. CBMC surface expression of CD69 was largely dependent on direct contact with fibroblasts. This suggests that interaction with subepithelial cells is important in MC activation.
Kang et al., 2024 [28]	Secreted phospholipase A2 Group IB (sPLA2GIB)	Human samples of NPs *plus* nasal epithelial cell cultures	Quantitative PCR, immunofluorescence staining, Western blotting, and enzyme-linked immunosorbent assay (ELISA)	Expression of sPLA2GIB (mainly in epithelial cells) was higher in eosinophilic CRSwNP than in control tissues. sPLA2GIB induced PGD2 and IL-13 production in human mast cells.
Kim et al., 2017 [29]	Periostin	Human samples of NPs *plus* human mast cell and bronchial cell cultures	Quantitative PCR, immunofluorescence staining, Western blotting, and enzyme-linked immunosorbent assay (ELISA)	Periostin is mainly produced by MCs in NPs. Periostin enhances TSLP secretion, which induces Th2 inflammation via dendritic cells. Periostin may be a novel biomarker for eosinophilic NPs, and that periostin inhibition may offer a new treatment strategy.
Kim et al., 2020 [30]	IL-22	Human samples of NPs *plus* human mast cell cultures	Quantitative PCR, immunofluorescence analysis	IL-22 expression was not higher in NP compared with controls. However, IL-22 positively correlated with type 2 immune mediators and the disease severity in NP. Eosinophils and MCs were important sources of IL-22. IL-22 receptor subunit alpha-1 (IL-22Ra1) expression was also significantly increased in NP.
Lee et al., 2021 [31]	Sialoglycan-binding lectin (Siglec-8)	Nasal lavages and excised surgical samples	Immunofluorescence and immunohistochemical analysis	Siglec-8 is overexpressed in eosinophils and MCs from CRSwNP patients. Deleted in Malignant Brain Tumors-1 (DMBT1) is an endogenous protein that carries O-linked sialylated keratan sulfate chains (S8) that inhibits MCs and eosinophil functions by binding to Siglec-8 (S8L). The S8L/DMBT1 ratio was significantly increased in CRSwNP vs. control or CRS patients. S8L expression was specifically elevated in the submucosal glands and epithelium of polyp tissue compared to middle turbinate. DMBT1 S8 (S8L) is produced in the submucosal glands of nasal polyps and secreted into the lumen of the sinonasal cavity; it may represent a host response to mitigate eosinophil-mediated inflammation.
Nagarkar et al., 2013 [32]	Thymic stromal lymphopoietin (TSLP)	Surgical samples of NPs	ELISA and Western blotting and activation bioassay	TSLP expression was significantly decreased in NP tissue compared to healthy and CRSsNP controls. NP extract-treated TSLP had higher activity in MCs. NP extracts significantly increased IL-1β-dependent IL-5 production in MCs, and responses were significantly inhibited by anti-TSLP antibodies.
Moon et al., 2014 [33]	DP2 (receptor for prostaglandin PGD₂, also known as CRTh2)	Surgical samples of NPs and bone marrow and nasal epithelial cultures	Immunohistochemistry and flow cytometric evaluation	Around 34% of MCs in human nasal polyps expressed DP2. Degranulation was not affected by DP2 selective agonists. Human MCs express DP2 intracellularly, whereas eosinophils and basophils use it as a surface receptor that induces Th2-related pathways.
Sugawara et al., 2013 [34]	Cannabinoid receptor 1 (CB1)	Serum-free NPs organ culture model	Immuno-histomorphometry and electron microscopy	Kit(+) human MCs express functional CB1 receptor in situ. Blockage of CB1 signaling induces MC degranulation. MC activation and maturation can be inhibited through CB1 stimulation.
Shin et al., 2015 [35]	Interleukin 25 (IL-25)	Surgical samples of NPs *plus* Murine model of nasal polyps	Immunohistochemistry, quantitative RT-PCR, and ELISA	IL-25 is upregulated in NP in comparison to healthy controls and with CRSsNP cases. MCs are a main source of IL-25 in humans. IL-25 mRNA expression was associated with other inflammatory markers. In mice, anti-IL-25 treatment reduced the formation of NPs.
Tóth et al., 2018 [36]	Neuronal Transient Receptor Potential Vanilloid 1 (TRPV1) and Ankyrin 1 (TRPA1)	Biopsy samples from patients versus controls	Quantitative PCR, protein localization by immunohistochemistry, and cytokine profile by multiplex bead immunoassay	Mast cells and macrophages expressed TRPV1 and TRPA1 receptors in NP. These receptors were not present on eosinophils. TRPV1 mRNA levels were significantly increased in CRSwNP patients with asthma and allergic rhinitis compared to controls.
Wang et al., 2017 [37]	Kynurenine (KYN)/aryl hydrocarbon receptor (AhR) and oxidized calmodulin-dependent protein kinase II (ox-CaMKII)	Surgical samples from patients versus controls	Quantitative PCR, immunohistochemistry, Western blotting, and mouse bone marrow-derived mast cells	NPs had increased expression of KYN compared with controls. KYN potentiated reactive oxygen species generation, cell activation, and expression of ox-CaMKII in wild-type, but not in AhR−/− MCs. Mast cells from ROS-resistant mice or pretreated with ox-CaMKII inhibitors showed reduced KYN-mediated MC activation.
Zhai et al., 2018 [38]	Immunoglobulin D (IgD)	Biopsy samples from patients versus controls	Quantitative PCR, protein localization by immunohistochemistry and cytokine profile by multiplex bead immunoassay	Expression of IgD heavy constant genes was higher in NP samples than controls. IgD+ plasmablasts were increased in both eosinophilic and noneosinophilic NPs, while IgD+ MCs were increased in eosinophilic polyps only. IgD stimulated polyp MCs to produce IL-21, IL-4, and IL-13. In eosinophilic polyps, expression of those B cell-stimulating factors in mast cells and close contact between mast cells and B cells were found. Moreover, positive correlations of total IgD levels with total IgE levels and eosinophilia and upregulation of specific IgD against house dust mites were discovered in eosinophilic polyps.
Zhai et al., 2019 [39]	CD30L/CD30	Biopsy samples from patients versus controls	Quantitative PCR, protein localization by immunohistochemistry and cytokine profile by multiplex bead immunoassay	CD30 was upregulated in eosinophilic NPs. MCs accounted for 60% of CD30L+ cells in eosinophilic NPs. CD30 induced HMC-1 cells to produce IL-4 and IL-13.
Zoabi et al., 2019 [40]	CD48	Biopsy samples from patients	Immunohistochemistry and flow cytometric evaluation	Membrane CD48 expression on eosinophils infiltrating allergy^NEG^ asthma^POS^ NPs was highly elevated in comparison to the other subgroups. CD48 and its high-affinity ligand m2B4’s expression on eosinophils from enzymatically digested NPs were significantly higher in allergy^NEG^ asthma^POS^ in comparison to allergy^POS^ asthma^POS^.

**Table 2:** A synthesis of the latest findings on MCs and CRS in the last 10 years by single-cell analysis techniques.

Reference	Type of Analysis	Sample	Main Findings
(Dwyer et al., 2021) [9]	scRNA-seq (seq-Well)	CRSwNP (Ethmoid sinus tissue, n = 6)	MC expansion is proportional to disease severity. MCs in the polyp environment can produce proteases, lipid mediators, and histamine. Two main subclusters of MCs were identified: MCs expressing tryptase and chymase (MC_TC_) and MCs expressing only tryptase (MC_T_). Both populations arise from the same common intermediate progenitor that is shaped by the tissue microenvironment. MC_TCs_ are poised to coordinate pro-inflammatory response through chemokine production, myeloid growth factor expression, IL13, and PTGS2. MC_Ts_ can potentially regulate a type 2 inflammation-driven response.
(Ordovas-Montanes et al., 2018) [41]	scRNA-seq (seq-Well)	CRSsNP (ethmoid sinus, n = 6) CRSwNP (ethmoid sinus, n = 6)	MCs and eosinophils are found only in polyps’ samples. MCs are generally enriched in HPGDS and PTGS2, which are involved in prostaglandin D2 production. A great number of MCs express IL5 and IL13.
(Xu et al., 2023) [42]	10x Genomics	Healthy control (inferior turbinate, n = 4) CRSwNP (polyp, n = 4; inferior turbinate, n = 4)	Mast cells are increased in nasal polyps compared to healthy control samples. No significant differences between nasal polyps and paired inferior turbinate tissue. No further characterizations were reported in the paper.
(Wang et al., 2022) [43]	10x Genomics	Healthy control (sphenoid sinus, n = 5) CRSsNP (ethmoid sinus, n = 5) eCRSwNP (ethmoid sinus, n = 6) neCRSwNP (ethmoid sinus, n = 6)	Two clusters of different mast cells were identified: CMA1^+^ MCs and APOE^+^ MCs. APOE^+^ MCs can be categorized among MC_Ts_, while CMA1^+^ can be categorized into MC_TCs_. The APOE+ subtype expresses high levels of pro-inflammatory receptors and genes associated with the metabolism of arachidonic acid. This subset can produce prostaglandin D2, leukotrienes, histamine, and nitric oxide. CMA1^+^ MCs are significantly more abundant in healthy controls, while APOE^+^ MCs are enriched in eCRSwNP tissues.
